# Finding novel distinctions between the sAPPα-mediated anabolic biochemical pathways in Autism Spectrum Disorder and Fragile X Syndrome plasma and brain tissue

**DOI:** 10.1038/srep26052

**Published:** 2016-05-23

**Authors:** Balmiki Ray, Deborah K. Sokol, Bryan Maloney, Debomoy K. Lahiri

**Affiliations:** 1 Indiana University School of Medicine, Departments of Psychiatry, Indianapolis, Indiana-46202, USA; 2 Indiana University School of Medicine, Departments of Neurology, Indianapolis, Indiana-46202, USA; 3 Indiana University School of Medicine, Departments of Medical and Molecular Genetics, Institute of Psychiatric Research, Indianapolis, Indiana-46202, USA; 4 Indiana Alzheimer Disease Center, Indianapolis, Indiana-46202, USA

## Abstract

Autism spectrum disorder (ASD) and Fragile X syndrome (FXS) are developmental disorders. No validated blood-based biomarkers exist for either, which impedes bench-to-bedside approaches. Amyloid-β (Aβ) precursor protein (APP) and metabolites are usually associated with Alzheimer’s disease (AD). APP cleavage by α-secretase produces potentially neurotrophic secreted APPα (sAPPα) and the P3 peptide fragment. β-site APP cleaving enzyme (BACE1) cleavage produces secreted APPβ (sAPPβ) and intact Aβ. Excess Aβ is potentially neurotoxic and can lead to atrophy of brain regions such as amygdala in AD. By contrast, amygdala is enlarged in ASD but not FXS. We previously reported elevated levels of sAPPα in ASD and FXS vs. controls. We now report elevated plasma Aβ and total APP levels in FXS compared to both ASD and typically developing controls, and elevated levels of sAPPα in ASD and FXS vs. controls. By contrast, plasma and brain sAPPβ and Aβ were lower in ASD vs. controls but elevated in FXS plasma vs. controls. We also detected age-dependent increase in an α-secretase in ASD brains. We report a novel mechanistic difference in APP pathways between ASD (processing) and FXS (expression) leading to distinct APP metabolite profiles in these two disorders. These novel, distinctive biochemical differences between ASD and FXS pave the way for blood-based biomarkers for ASD and FXS.

Autism spectrum disorder (ASD) is a developmental disorder with a complex etiology. It is characterized by persistent deficits in social communication and social interaction across multiple contexts and restricted, repetitive patterns of behavior, interests, or activities. Symptoms must be present in the early developmental period and the disturbances are not better explained by intellectual disability^1^. Fragile X Syndrome (FXS) is often but not always associated with autism-like behavioral symptoms but also is associated with intellectual disability, macroorchidism, macrocephaly (shared with some ASD cases), and seizures (shared with some severe ASD cases). FXS is caused by the expansion of a CGG trinucleotide repeat in the 5′ untranslated region of the fragile X mental retardation 1 (*FMR1*) gene located in the X chromosome^2^,^3^,^4^ or by deletion of *FMR1*^3^. FXS is the most common type of inherited cognitive disability, affecting approximately 1 in 4000 males and 1 in 8000 females and it is the most prevalent known genetic correlate of autism-like symptoms^5^. Brain abnormalities common to FXS and ASD are deranged cortical pyramidal dendritic spines^6^,^7^ and increased disruption of white more than gray matter pathways^8^,^9^ contributing to macrocephaly, seen in both these disorders.

Amyloid-β precursor protein (APP) is a large (695–770 amino acid) membrane spanning glycoprotein expressed ubiquitously in brain^10^,^11^. Predominantly located at synapses, APP is axonally transported and secreted from axon terminals in response to synaptic activation^12^. Regardless of its necessity in normal neurological development and maintenance, APP is most often mentioned in association with Alzheimer’s disease (AD), the most prevalent dementia^13^, affecting 1 in 6 women and 1 in 11 men over the age of 65^14^. AD brain pathology is characterized by neuronal death, neurofibrillary tangles consisting primarily of hyperphosphorylated microtubule-associated protein τ, and amyloid plaques consisting primarily of neurotoxic amyloid-β peptide (Aβ). The AD-associated “amyloidogenic” pathway favors cleavage of APP by β-secretase (BACE1), resulting in the 40 and 42 amino acid Aβ and soluble APPβ (sAPPβ). Aβ peptides are major components of the extracellular cerebral amyloid plaques associated with brain atrophy found in AD. Alternatively, the proteolytic cleavage of APP via the α-secretase pathway releases neurotrophic sAPPα^12^,^15^ (Fig. 1). An excess in this pathway (sAPPα and low Aβ) may result in a gain of function overgrowth and anabolic state associated with neurodevelopmental conditions^16^, while the expected plasma neuronal marker profile in AD is low sAPPα and high Aβ. Our goal is to provide a biochemical basis to explain common features and differences between ASD and FXS regarding neurobiology, neuroanatomy, and behavior. In two independent studies^15^,^17^, our group has reported higher sAPPα levels and lower Aβ peptide levels in plasma (n = 26) and brain tissue (n = 7)^18^ of children with severe autism. The sAPPα results have been replicated by an independent laboratory^19^,^20^. Recently, we found increased sAPPα, APP, and Aβ plasma markers in children with FXS (n = 12) compared to youth with ASD (n = 11)^21^. This study was limited by lack of a typically developing control group, which we have included in the present study. However, there is no publication of levels of different APP metabolites in brain tissue specimens. We now report a consistent increase in protein levels of sAPPα accompanied by decreased sAPPβ and Aβ in ASD vs. neurotypical controls. By contrast, we found an increase in levels of sAPPα, sAPPβ, and Aβ in FXS vs. neurotypical controls in the few brain tissue samples available to us. These differences are significant between ASD and FXS.

Behavioral and gross anatomical (macrocephaly) similarities are well known between ASD and FXS; however, multiple behavioral, finer anatomical, and biochemical differences are emerging. Notably, neuroanatomical differences have been reported in brains of ASD and FXS subjects. For example, while the caudate nucleus is enlarged in both FXS and ASD^22^,^23^, the amygdala is reduced in FXS and enlarged in ASD^24^ to the extent that the amygdala in ASD is significantly larger than in FXS^25^. Interestingly, even though enlarged in both conditions, the caudate nucleus in FXS subjects is significantly larger than in ASD. Contrasting these two disorders against AD, we note a report showing a significant atrophy of the amygdala in early AD, which relates to symptom severity^26^. On the other hand, enlargement of the amygdala correlated with social and communication deficits on the Autism Diagnostic Interview-Revised (ADI-R) in idiopathic autistic children^27^. We are tempted to draw a preliminary hypothesis supported by negative analogy, i.e., amygdala enlargement in ASD vs amygdala atrophy in AD. By neurobiological corollary hypothesis, an “Aβ deficit” (opposite to “Aβ excess” in AD) may operate in concert with the neurotrophic functions of sAPPα to produce features unique to ASD vs. FXS.

Other cellular pathways for ASD and FXS are also consistent with our hypothesis. At the molecular level, FMRP regulates many messenger ribonucleic acids (mRNAs) via signal transduction, including APP mRNA^28^. Evidence from animal studies^28^,^29^,^30^,^31^,^32^ suggests that FMRP mRNA, required for metabotropic glutamate receptor (mGluR) translation of synaptic proteins, is linked to APP mRNA via a G-quartet-like motif coding region, thereby establishing a link between FXS and AD^28^. Overexpression of both APP and Aβ was associated with seizures in an FXS mouse model^28^,^29^. Removal of one APP allele reversed that FXS phenotype and reduced Aβ levels to normal^30^. Given trophic actions of APP and sAPPα, the mGluR5 response may act as a master switch that balances catabolic and anabolic processes in nervous system development^33^,^34^. In previous models^33^, decreased FMRP favored loss of normal constraints on synaptic activity-induced protein synthesis (producing a net increase in APP and sAPPα cleavage product), leading to the FXS-like and autism-like symptoms in the animal.

In short, we here present data that refines the sAPPα-based anabolic model to not only explain shared symptoms of ASD and FXS, but also begin to shed light on neurochemical and neurobiological differences between the two conditions, particularly differences that may likewise explain previously reported neuroanatomical and behavioral contrasts between ASD and FXS. We unite our findings with other distinctions between ASD and FXS to illustrate how they overlap and differ at neurochemical, biological, neuroanatomical, behavioral and psychiatric levels.

## Results

### APP metabolic products in ASD and FXS plasma

#### Model Selection

All analyses tested potential covariates, specifically age and IQ. The second-order Akaike information criterion (AICc)^35^ was used to choose suitable models. AICc indicated that biomarker ~ diagnosis was the preferred model for all plasma samples.

#### Soluble APP (sAPP)

**Figure 2:**
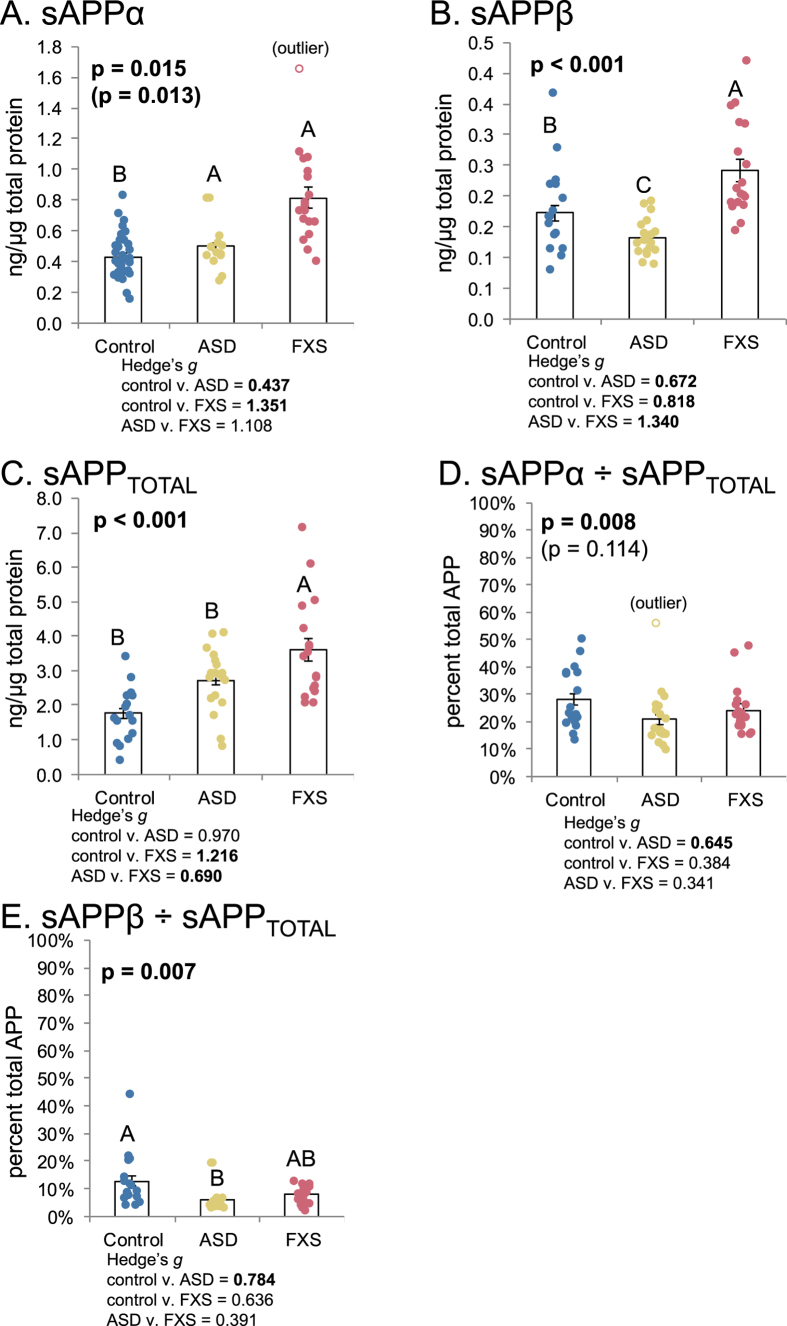
Levels of various Soluble APP (sAPP) isoforms in ASD and FXS plasma. Plasma from age-matched controls, autistic subjects, and subjects with Fragile X was collected and albumin-subtracted as described in the text. ELISA was performed for sAPPα, sAPPβ and total sAPP in plasma. Generalized linear models (glm) were run as described in the text and ANOVAs run on the glm vs. a null model. Each model was subject to a Bonferroni-corrected test for outliers, and glm, repeated omitting any outlier. The p value(s) for any glm including the outlier are in parentheses. Each glm with p ≤ 0.05 was followed by simultaneous pairwise comparisons (Tukey’s). Diagnoses sharing a letter do not significantly differ. Hedge’s *g* standardized effect sizes were also calculated for each pairwise comparison. Specific *g* are reported beneath each chart. Comparisons with Tukey’s p ≤ 0.05 are in boldface. (**A**) sAPPα levels. FXS and ASD significantly differ from controls. (**B**) sAPPβ levels. FXS is significantly higher than control. ASD is significantly lower than control. (**C**) Total APP levels in plasma. Overall, controls have lower levels of total measured APP than other groups, but this difference is not significant for control vs. ASD. **(D**) sAPPα/APP_TOTAL_. While the trimmed (outlier-excluded) model had overall significance, no pairwise comparisons were significant. (**E**) sAPPβ/APP_TOTAL_. Scale at 0–100% for direct comparison. ASD showed significantly lower levels of this ratio than controls. FXS overlapped the other two groups.

We measured plasma levels of different forms of soluble APP (sAPP) with three independent assays. These were sensitive sandwich enzyme-linked immunosorbent assays (ELISA) specific for levels of sAPPα, sAPPβ, and all sAPP isoforms (sAPP_TOTAL_). Each ELISA used different peptide end-specific capture and detection antibodies. Data were analyzed by “sAPPn ~ Diagnosis”, where “sAPPn” refers to the appropriate form of sAPP. Diagnoses were “control”, “ASD”, and “FXS”. Two trends emerged when looking at sAPPn (Fig. 2). Levels of sAPPα (Fig. 2A) were significantly higher in ASD and FXS than in control samples. Levels of sAPPβ (Fig. 2B) were distinct among diagnoses: ASD < control < FXS. Levels of sAPP_TOTAL_ (Fig. 2C) were less distinctly divided. In particular, only FXS significantly differed from controls. In short, while sAPP_TOTAL_ increases in FXS were accompanied by increases in both sAPPβ and sAPPα, levels of sAPPβ in ASD were reduced in comparison to not only FXS but also controls. Adjusting sAPPα and sAPPβ by sAPP_TOTAL_, however, obscured this processing difference (Fig. 2D–E). Adjusting sAPPα ELISA by sAPP_TOTAL_ ELISA (Fig. 2D) greatly reduced differences between groups, initially suggesting that variations in sAPPα levels due to ASD or FXS may be due to differences in *APP* gene expression. However, adjusting sAPPβ ELISA results by sAPP_TOTAL_ presents a somewhat different picture. While no significant differences exists between control and FXS, ASD shows a significant decrease vs controls, which mirrors the significant decrease found for unadjusted sAPPβ, although ASD and FXS ratios are not different. Notably, this suggests differences in APP processing pathway regulation between ASD and FXS: Abnormal APP processing in ASD (decreased sAPPβ/sAPP_TOTAL_) or of APP expression in FXS (unchanged sAPPβ/sAPP_TOTAL_) vs controls.

#### Amyloid β (Aβ) peptides

**Figure 3:**
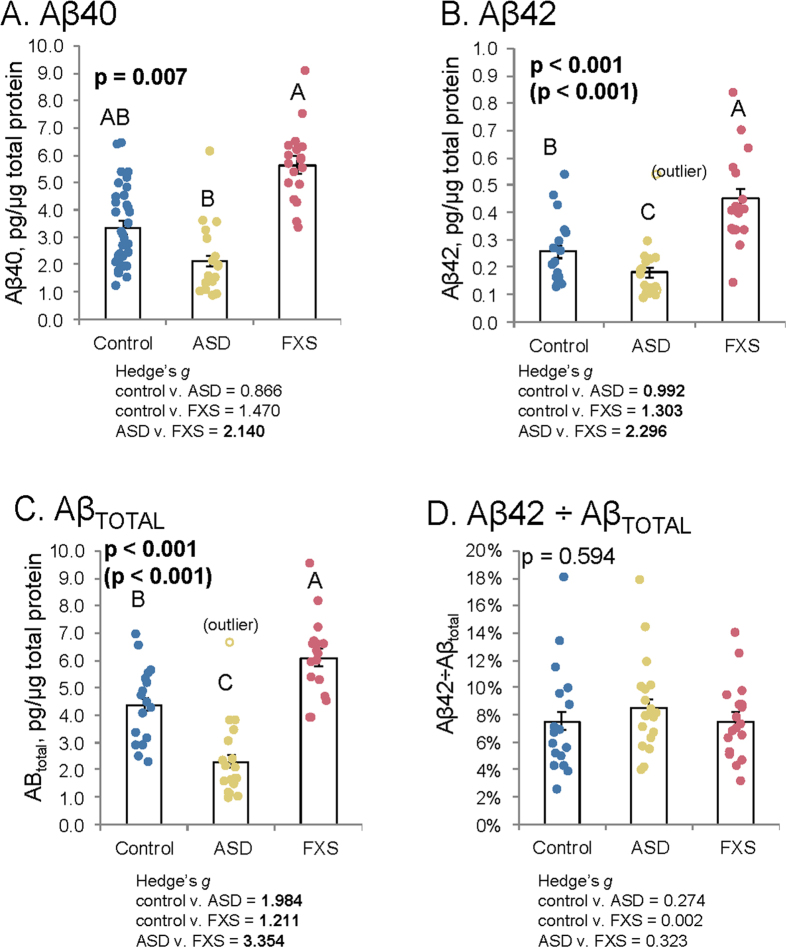
Amyloid β (Aβ) in ASD and FXS plasma. Plasma from age-matched controls, autistic subjects, and subjects with Fragile X was collected and albumin-subtracted as described in the text. ELISA was performed for Aβ40 and Aβ42 in plasma. Generalized linear models (glm) were run as described in the text. Each model was subject to a Bonferroni-corrected test for outliers, and glm repeated omitting any outlier. Each glm with p ≤ 0.05 was followed by simultaneous pairwise comparisons (Tukey’s). Diagnoses sharing a letter do not significantly differ. Hedge’s *g* standardized effect sizes were also calculated for each pairwise comparison. Specific *g* are reported beneath each chart. Comparisons with Tukey’s p ≤ 0.05 are in boldface. (**A**) Aβ40 levels. FXS samples had highest levels, but did not significantly differ from controls. ASD showed significantly lower levels of Aβ40 from FXS but not controls. (**B**) Aβ42 levels. FXS had elevated levels of Aβ42. ASD showed significantly lower levels of Aβ42 than controls. (**C**) Total Aβ levels. Combining levels of Aβ40 and Aβ42 accentuated differences seen for each individual form. Specifically, both FXS had highest levels of Aβ, significantly exceeding control and ASD was significantly lower than control and FXS. (**D**) Aβ42 proportion. Derived from Aβ42 ÷ (Aβ42 + Aβ40). No significant differences found in specific types of Aβ processing.

We also performed ELISA to measure levels of amyloid β (Aβ) peptides, both 40-amino acid (Aβ40) and the 42-amino acid (Aβ42) species (Fig. 3). Levels of Aβ40 were different between ASD and FXS, but controls were not significantly different from either condition (Fig. 3A). Aβ42 levels were more distinct: ASD < control < FXS (Fig. 3B). Combining Aβ40 and 42 (Fig. 3C) produced the same trend as Aβ42 alone. Aβ processing by ratio of Aβ42 to Aβ_TOTAL_ showed no significant differences (Fig. 3D).

### APP metabolic products in ASD and FXS brain tissue samples

#### Model Selection

All analyses tested potential covariates, specifically age and IQ. The second-order Akaike information criterion (AICc)^35^ was used to choose suitable models. AICc indicated that biomarker ~ diagnosis was the preferred model except for β actin-adjusted A Disintegrin and Metalloproteinase Domain 17 (ADAM17) (also known as “tumor necrosis factor-α converting enzyme” or TACE), which was instead compared to “diagnosis + age + diagnosis × age”. Models with age covariate were also run for the other biomarkers in the study (Supplemental data) but were not used for analysis. In addition, low FXS sample size vs. two factors plus an interaction, as opposed to a single factor, would present a risk of overfitting the models.

#### Biomarker levels by diagnosis

**Figure 4:**
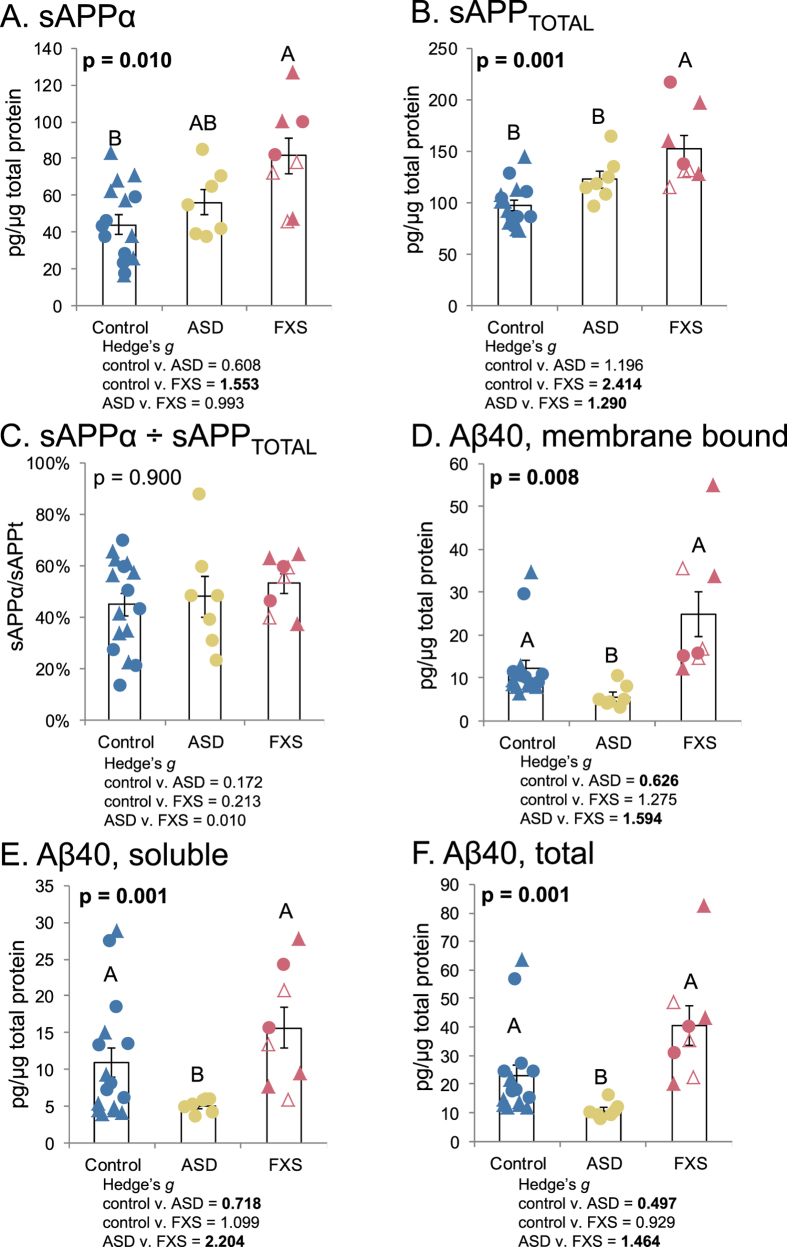
APP and Aβ40 levels in ASD and FXS brains. Brain samples were obtained as described in the text. Samples were processed and subject to Western blotting or ELISA as described in the text. In some cases, two brain regions were used for FXS samples, BA21 indicated by solid markers, BA10 indicated by hollow markers. Triangles indicate samples used in the two-brain-region assay. Samples were analyzed either by generalized mixed models (glmm), followed by ANOVA comparing the model with a null model. The glmm was used to take account for assay and brain region as random variables. Models were tested for outliers by Bonferroni-corrected method. No outliers were found in the models. Models with p ≤ 0.05 were further subject to simultaneous pairwise comparisons (Tukey’s). Letters indicate pairwise categories. (**A**) sAPPα levels differed between control and FXS, while ASD was intermediate between the two. (**B**) Total APP levels fell into two categories, with control being significantly lower than FXS, but no significant difference between control and ASD. (**C**) The ratio of sAPPα to total APP did not differ among diagnoses. (**D**) Levels of DEA-extracted (membrane-bound) Aβ were significantly lower in ASD samples than in either control or FXS. (**E**) Levels of soluble Aβ were significantly lower in ASD samples than in either control or FXS. (**F**) Levels of total (soluble + membrane-bound) Aβ40 per diagnosis.

Brain frontal cortex (regions BA10 and BA21) samples of control (n = 8), ASD (n = 7) and FXS (n = 3 adult subjects including one with FXS pre-mutation) subjects were assayed for levels of sAPP_TOTAL_, sAPPα, Aβ40, and membrane-bound Aβ40. Analyses were done with mixed models. Aβ40 extraction was done twice from samples, first to extract soluble Aβ40, then a second extraction was done from the pellet remnant to extract membrane-associated Aβ40. Significant differences were found among diagnoses for sAPPα (Fig. 4A) and sAPP_TOTAL_ (Fig. 4B). Levels of sAPPα increased from control < ASD < FXS, but only the difference between control and FXS was significant. For sAPP_TOTAL_, FXS was significantly higher than both control and ASD. Comparing the ratio of sAPPα ÷ sAPP_TOTAL_ produced levels that were very close for all three diagnoses (4C). Aβ40 and soluble Aβ40 had the same general profile (4D–F), specifically, that ASD samples had significantly lower levels of Aβ40 than did either control or FXS, while control and FXS were not significantly different from each other. This carried over to total Aβ40 (soluble + membrane bound).

### ADAM17 α-secretase levels vs. age in ASD vs. controls

**Figure 5:**
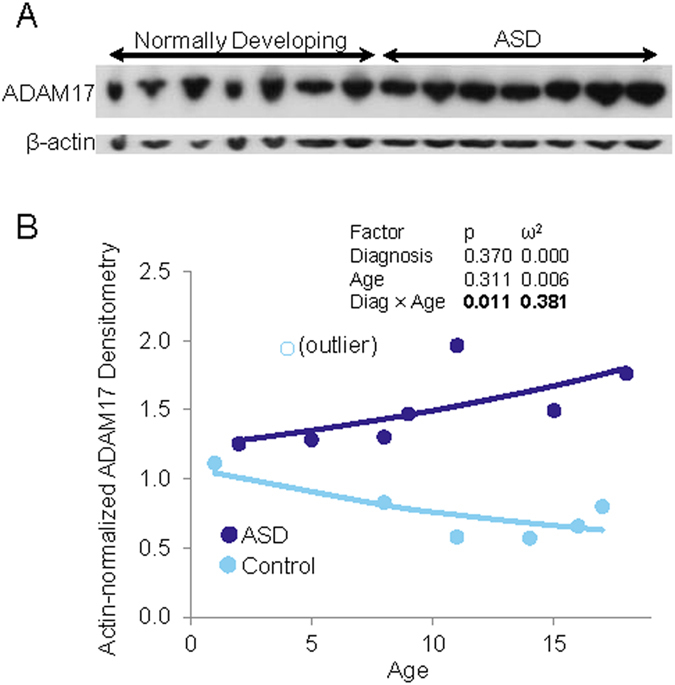
ADAM17 levels in ASD depend on both diagnosis and age. (**A**) Brain samples were obtained as described in the text. Control and ASD samples were processed, run on denaturing SDS-PAGE, and subject to Western blotting for ADAM17. All samples were run under the same conditions in one gel. The blot was cut into two parts: The top part was probed with anti-ADAM-17 and the bottom part with anti-β-actin antibodies. No lane was cut, cropped, merger or modified in any way. (**B**) Western blot was densitometrically scanned, and ADAM17 densitometry signal was adjusted for β-actin densitometry. A two-way glm of ADAM 17 ~ (Diagnosis + Age)^2^ was tested. The interaction of Diagnosis × Age was found significant. A single outlier control was detected by Bonferroni-adjusted test and modeling was repeated without this outlier. Results of outlier-excluded modeling are shown. Control and autistic individual samples are shown along with model-fitted lines. Age corresponds to increasing levels of ADAM17 in autistic subjects while age corresponds to decreasing levels of ADAM17 in control subjects.

Comparing β actin-adjusted ADAM17 to a two-way model of diagnosis and age, with interaction predicted a significant interaction between control/ASD status and age (Fig. 5). In control samples, adjusted ADAM17 signal decreased with age, while adjusted ADAM17 levels increased with age in ASD (Fig. 5B). All subjects were age 18 or younger. Unfortunately, insufficient samples were available to include FXS in the model.

## Discussion

The growing number of genes associated with autism and autism symptoms affect neuronal activity across three networks^4^: 1) “local” dendrite signaling that controls synapse connection and function 2) regulation of post-synaptic dendritic mRNA translation, underlying synaptic plasticity, and 3) synaptic modification by nuclear gene transcription. Synaptic plasticity, in which the brain encodes and stores information, depends upon persistent, activity-dependent changes at individual synapses, synaptic elimination, and long-term potentiation (LTP) and the opposing process, long term depression (LTD)^36^. These processes are regulated via ionotropic^37^ and metabotropic^38^ receptors. For example, glutamate receptor-mediated synaptic plasticity involving LTD is associated with synaptic elimination in brain development^39^. Soluble APP forms, particularly sAPPα, take part in this synaptic plasticity modeling and remodeling^12^,^40^.

Soluble APPα has a likely role in FXS and ASD^41^. There is overlap among APP pathways, FMRP targets, and several autism-associated genes across the three networks. “Local” synaptic activity is critical to the development of autistic features: Adaptive quality of the dendritic spines provides the basis for synaptic plasticity^4^,^42^. The most-studied structural abnormality in FXS and *Fmr1* knock out (KO) mice is increased density of immature dendritic spines^43^,^44^, These, too, have been reported in the brains of ASD human subjects^6^. FMRP deficiency has been shown to affect pre- and postsynaptic contacts. Studies utilizing *Drosophila* FXS model show an abundance of immature mini/satellite boutons suggesting that FMRP has a primary role in restricting bouton deposition. Similarly, *Drosophila* lacking APP show reduced boutons, while overexpression of APP increases bouton formation^45^. This suggests a role for both FMRP and APP at the synapse. Adhesion molecules and the post-synaptic density (PSD), a region of structural proteins that signal molecules and receptors in the dendritic spine head, regulate excitatory synapses and stimulate activity-dependent signaling networks within the post synaptic neuron^4^. Synaptic cell adhesion molecules anchor the presynaptic vesicle to the PSD. Cell adhesion genes (cadherins and neuroligins) direct this pathway; catenins reside in the cadherin protein complex that form synapse adherens junctions.

Recent findings show that, 39% (49 of 126) of the most severely disrupted *de novo* autism-associated mutations map to a β-catenin/chromatin pathway^46^. Disruption of the β-catenin pathway may lead to macrocephaly via faulty cell adhesion, favoring release of tight synaptic coupling, which otherwise reduces growth of brain progenitor cells^47^,^48^. In support of this, rare mutations in several neurexin and neuroligin synaptic adhesion molecules have been associated with autism^49^. FMRP also modulates growth, although the direction of growth may be influenced by intermediary factors. FMRP affects the expression of selected mRNAs, for example, either by enhancing mRNA stability or by blocking its translation^50^,^51^. FMRP contributes to excitatory synapse elimination in mouse neurons, working together with the activity-dependent transcription factor myocyte enhancer factor 2 (*mef2*) and protocadherin 10 (*pcdh10*), the human homologue associated with autism, and a member of the cadherin complex^52^. It is speculated that lack of FMRP in FXS^52^ and disruption of *mammalian target of rapamycin* (mTOR) autophagy in autism^53^ favor decreased synapse elimination consistent with anabolism and macrocephaly. In addition to its loss in FXS, FMRP levels are decreased in brain samples from autistic subjects^54^. Further, FMRP binds to Down syndrome cell adhesion molecule (Dscam) RNA, involved in neural development^55^.

FMRP is expressed in organs other than brain; FMRP binds adhesion molecules such as E‐cadherin mRNA, resulting in the enhancement of breast and lung cancer *in vitro*^56^. Interestingly, lack of FMRP on cell adhesion in FXS may result in the lower incidence of cancer seen in individuals with FXS^56^,^57^. Likewise, studies of conserved domains support an adhesion property for all APP metabolites^58^. APP directly induces presynaptic adhesion similar to that of neurexins and neuroligins^59^ and interacts with other adhesion molecules (integrins^60^ and β-catenin^61^). Long lasting synaptic plasticity occurs during gene activity when glutamate binds to N-Methyl-D-Aspartate (NMDA) or mGluR receptors. Polyribosomes collect near synapses where protein synthesis is mediated by mRNAs^4^. FMRP shuttles from nucleus to the cytoplasm where it interacts with polyribosomes, binding to and inhibiting dendritic translation of many brain mRNAs including APP^29^. APP is regulated by FMRP via the mGluR receptor^29^.

APP and its metabolites also have a purported anabolic role within other translation regulating pathways such as Ras small GTPase/Extracellular signal-Regulated Kinase (Ras/ERK)^62^, phosphoinositide 3 kinase (PI3K)/mTOR^63^, and key signaling modulator Striatal-Enriched Protein Tyrosine Phosphatase (STEP)^64^. Several genes that often occur in minority polymorphisms in autism (*FMR1*, Tuberous Sclerosis complex 1 and 2 -*TSC1, TSC2*, and phosphatase and tensin homolog-*PTEN*) and the STEP protein that is upregulated in FXS^65^ are associated with these pathways. Finally, synaptic transmission is strengthened via LTP and weakened via LTD. mGluR -LTD is exaggerated in the mouse *Fmr1* KO hippocampus^66^, supporting FMRP’s role in synaptic plasticity.

Aβ is not automatically pathogenic and is likely to have multiple normal functions, including regulation of metal homeostasis, normal inflammatory response, and function as a transcription factor^67^. On the other hand, excessive Aβ is acutely associated with decreases in LTP, learning, and memory^68^, and chronically associated with neurodegeneration while sAPPα is associated with increased LTP and spatial memory^69^. APP may also contribute to gene network effects in ASD and FXS that are multiple steps away from dendritic growth and function^4^. FXS is caused by the complete transcriptional shutdown or loss of the *FMR1* gene, while the pre-mutation disease, fragile X-associated tremor/ataxia syndrome (FRAXTAS), results from excess repeat-containing RNA transcripts^70^. Essentially, mutations in *FMR1* cause two significantly different transcriptional defects/disorders. We propose that dysregulation in the APP mRNA gene similarly can result in different disorders: AD and autism.

The inflammatory-responsive transcription factor specificity protein 1 (Sp1) is required for histone binding and gene activation. Sp1 activity is the strongest factor involved in *FMR1* transcription^70^, but its binding occupancy on the *FMR1* promoter is lost in FXS. Sp1 is a positive regulator of *APP*, as demonstrated by reports of DNA binding^71^, gene expression^72^, and deletion mutagenesis^73^. Elevated levels of Sp1 have been found in the frontal lobes of deceased patients with AD^74^; Sp1 has been associated with neuroinflammation in AD and other diseases such as autism. Sp1 was found to be elevated within the anterior cingulate gyrus (ACG) in brain tissue samples from children with autism^75^. Further, this autism study showed reduced reelin (*RELN*), a candidate autism gene with potential Sp1 binding site, in the ACG region. *RELN* has been associated with neurocognitive development^76^ and is part of the apolipoprotein E (apoE) biochemical pathway that is involved in the pathogenesis of AD^77^. Excessive Sp1 in FXS, autism, and AD implicates inflammatory reactions, which have been associated with each of these conditions. Finally, sAPPα has been shown to regulate gene expression by multiple transcriptional mechanisms such as nuclear factor (NF)-kB, cAMP response element binding protein (CREB), microRNA, and modulation of chromatin^78^. Through these mechanisms, sAPPα has been shown to down regulate apoptosis, favoring neurogenesis^78^. CREB has been shown to bind to the *FMR1* promoter^70^, and by activating brain-derived neurotrophic factor (*BDNF*) transcription controls the number of inhibitory synapse that form on excitatory neurons^79^. APP has been associated with BDNF protein^80^ that goes on to regulate synaptic formation, maturation, plasticity and elimination^81^. Further, overexpression of BDNF has been shown to induce myelination of active axons^82^ that may contribute to excessive white matter in autism. We^15^ and others^83^,^84^ have found abnormal levels of BDNF in plasma of children with autism. Further, sAPPα activates microglia, and oligodendrocytes^85^, which may increase white matter in autism.

Secreted APP metabolites are broadly increased in FXS plasma, which agrees with our previous study showing increased APP and Aβ plasma markers in children with FXS (n = 12) compared to children with ASD (n = 11)^21^. Increased APP and Aβ markers had also been found *in vitro*^28^ and in animal model studies^29^ demonstrating elevated APP expression in response to FMRP knockout. In contrast, sAPPα, but not Aβ is increased in ASD plasma^15^,^17^, suggesting that APP is preferentially processed by α-secretase. That is, we found elevation in APP and sAPPα and *decrease* in Aβ peptides in two independent plasma studies of children with autism^17^,^20^.

In our present brain tissue study, the reduction in Aβ peptides was accompanied by reduction in sAPPβ, making it more likely that alterations in the α-secretase vs. β-secretase processing of APP is the operating process in ASD, and not greater clearance of Aβ. Increased clearance of Aβ would result in no decrease of sAPPβ levels. We observed decrease of sAPPβ with Aβ in ASD samples. Levels of sAPPβ and sAPPα are not determined by Aβ clearance. Of greater interest is that plasma Aβ and sAPPβ levels were increased in FXS samples vs. both controls and ASD, along with sAPPα. Therefore, although APP markers appear to be peripherally dysregulated in both FXS and autism^15^,^17^ the conditions have distinct differences in their dysregulation. Reduced Aβ levels in plasma from autistic subjects (particularly Aβ42) may have potential diagnostic value. Westmark *et al*.^30^, reported lower levels of Aβ42 in adult FXS patient plasma, with no difference in APP_TOTAL_, sAPPα, or Aβ40 levels between FXS and control^30^. This was in transgenic mouse models, not human subjects diagnosed with ASD or FXS. Further studies are required to understand the consequence(s) of different Aβ levels in FXS and ASD individuals and perhaps to redefine practical limits of current animal models.

Interestingly, similar to our FXS plasma findings, total sAPP, sAPPα, and Aβ peptides were elevated in brain tissue of individuals with FXS. In contrast to FXS, but consistent with our plasma findings, we found definitely *decreased* Aβ peptides in brain tissue samples of children with ASD^18^. While our findings should be interpreted cautiously due to limited sample size, they raise potentially exciting prospects in understanding key aspects of sporadic vs. syndromic autism symptoms. Our plasma data was suggestive of differences in APP processing between ASD and FXS. Combination with our brain data is conclusive.

Diagnosis of “autism” among children with FXS is inconsistent and significantly varies according to which diagnostic (Autism Diagnostic Observation Schedule-ADOS, ADI-R, Diagnostic and Statistical Manual-DSM-IV) is used or if more than one is used^86^,^87^. Behavioral differences have also been found between ASD and FXS, specifically: In children of both sexes, impaired social behaviors (e.g., social smiling, range of social expressions, quality of social overtures, joint attention) and communicative behaviors (gestures, pointing, imitation), occurred at significantly lower rates for FXS than in ASD^88^,^89^. In addition, FMRP levels were not correlated to severity of FXS autistic symptoms if nonverbal IQ was taken into account^88^,^90^. Wechsler-tested IQ of FXS vs. ASD children (and parents) revealed that FXS subjects had, as a group, significantly lower IQ than ASD^89^. Likewise, no relationship was found between midparental and child IQ for FXS, while a significant relationship occurred for midparental vs child IQ in ASD^89^. Elevation of sAPPα and Aβ peptides may confer a “double deleterious dose” in FXS vs ASD. Alternatively, effects of elevating sAPPα and reducing Aβ may each be distinct.

Both ASD and FXS are complex disorders, and multiple biomarkers may contribute to their manifestation and progression^91^,^92^. The current diagnostic criteria for ASD in general include additional “clinical specifiers”, including but not limited to cognitive ability, association with other medical or genetic conditions, and deficiency in linguistic development, any or all of which may vary among confirmed ASD cases^1^. Any or all of these clinical specifiers may be associated with specific biomarker profiles^91^,^93^. The α-secretase (TACE) that cleaves sAPPα has been associated with increased oligodendrocyte growth^94^. Could increased sAPPα contribute to excessive increased brain white matter seen in both FXS and ASD?

**Figure 6:**
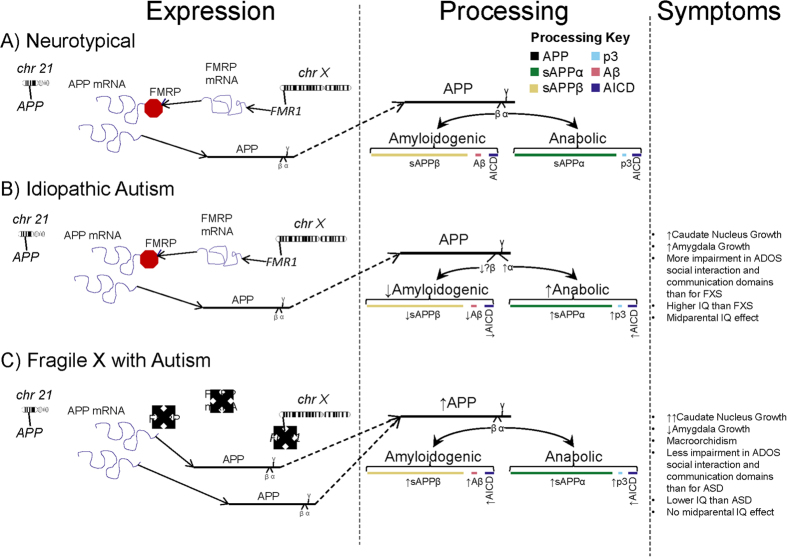
APP in ASD and FXS. An expansion of the anabolic model for autism-like symptoms. APP levels and metabolites can contribute to autism symptoms in both syndromic and idiopathic autism, although not necessary through identical pathways. (**A**) Neurotypical APP expression and processing involves partial downregulation of APP mRNA (transcribed from the *APP* gene on chromosome 21) by FMRP (from the *FMR1* gene on chromosome X). This results in normal levels of the APP protein, which is then processed in the amyloidogenic and anabolic pathways. (**B**) Idiopathic Autism. Idiopathic autism does not require lack of FMRP and can occur with normal levels of APP holoprotein. Once the holoprotein is produced, processing is tilted to abnormally favor the anabolic. This is likely to be through enhancement of α-secretase levels (such as ADAM17), but simultaneous deficiency of β-secretase cannot be ruled out. The result is reduction of amyloidogenic processing and increased anabolic processing, contributing to symptoms of ASD. (**C**) Fragile X deregulation of APP. In FXS, FMRP is absent. APP mRNA translation is not downregulated to normal levels, and excess APP protein is produced. This is processed through both pathways, resulting in greater levels of all APP metabolites. These contribute to some level or another to the various anatomical and behavioral symptoms of FXS. Overall increases of amyloidogenic products in addition to anabolic may explain some symptomatic differences between ASD and FXS. We do not rule out the possibility of a “mixed case” for some individuals, where a combination of impaired (but not absent) FMRP levels may combine with excess α-pathway processing to produce both excess APP and α-processing products.

As previously described, anatomical differences have been found in brains of FXS subjects and those with ASD. The caudate nucleus is enlarged in both FXS and ASD^22^,^23^. However, the amygdala is enlarged in ASD^24^, while it is reduced in FXS subjects^23^. Direct comparison of the two conditions revealed that the caudate nucleus in FXS subjects is significantly larger than in ASD *and* the amygdala in ASD is significantly larger than in FXS^25^. Thus, while there are some behavioral and gross anatomical (macrocephaly) similarities between ASD and FXS, multiple differences must be noted. To the behavioral and neuroanatomical comparisons, we add a neurobiological distinction. While ASD and FXS share elevated sAPPα (but not total sAPP), ASD has significantly reduced Aβ and sAPPβ. It is unlikely that this biochemical difference accounts on its own for all symptomatic divergence between FXS and ASD. On the other hand, enlargement of the amygdala correlated with social and communication deficits in ASD^27^. By negative analogy, early atrophy of the amygdala is related to severity in AD^26^. Thus, an “Aβ deficit” may operate in concert with the neurotrophic functions of sAPPα to produce features unique to ASD vs. FXS. This questions hypotheses that relied primarily upon FMRP and its regulation of mGluR5 to explain both FXS and ASD, but it does not exclude a role for FMRP/mGluR5 in ASD. Uniting our findings with other distinctions between ASD and FXS illustrates how they overlap and differ at neurochemical/biological, neuroanatomical, and behavioral/psychiatric levels (Fig. 6). One symptom that appears in some ASD and FXS cases is seizures. While our brain sample set did include some records of seizures, no information was available for controls and all FXS subjects below the age of 70 had recorded seizures. We cannot tell from our present sample how seizures may reflect the interplay among α- and β-secretase products vs. ASD and FXS. Localization of APP metabolites in ASD and FXS brains may also prove enlightening. Does anabolic sAPPα localize to white matter, known to be enlarged in ASD and FXS? Does Aβ localize to gray matter, associated with seizures and intellectual deficiency (if they appear) in these neurodevelopmental conditions? If so, then would reduced Aβ in ASD correlate to reduced risk of seizures vs. FXS? We hope to address these questions in future studies.

**Figure 7:**
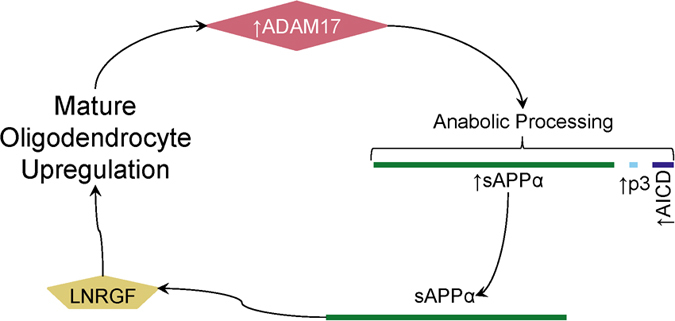
Anabolic APP processing feedback loop. One tentative model that may explain the contribution of sAPPα in an anabolic cascade relies on LNRGF activity in oligodendrocytes as an sAPPα receptor. ADAM17 is one of the α-secretases, which processes APP in the anabolic pathway, producing sAPPα. LNRGF is a receptor for sAPPα. LNRGF activation upregulates mature oligodendrocytes, which then produce additional ADAM17. A breakdown in regulation of this cycle may lead to out-of-control looping that produces excessive sAPPα, which contributes to autism symptoms.

In addition to these questions, sAPPα is a secreted protein that must interact with cells in order to influence neuroproliferation, neurite outgrowth, or other functions. Two known receptors or receptor-complex molecules for sAPPα are sortilin-related receptor L (DLR class) A repeats containing (SORL1)^95^ and low-affinity nerve growth factor receptor (LNGFR, aka p75NTR)^96^. SORL1 is implicated in regulating levels of Aβ^97^ and in the regulation of neuroproliferation and neuroprotection through the kinase cyclin dependent kinase 5 (CDK5). However, population studies revealed that CDK5 polymorphisms may not be associated with ASD^98^. When activity of LNGFR is examined, sAPPα binding to LNGFR promotes neurite outgrowth^99^. Of particular interest is that the valproic acid induction model of autistic behavior has both increased BDNF expression and doubled levels of LNGFR^100^. LNGRF upregulates mature oligodendrocytes^101^, and ADAM17 is expressed by oligodendrocytes during white matter myelination^94^. A putative pathway emerges wherein sAPPα production may be part of a feedback loop that could lead to increased white matter if loop regulation fails (Fig. 7). Corroborating this, enlarged brain volume, measured by MRI, occurs in autistic boys with increased levels of sAPPα^102^. We suggest that future work should explore the sAPPα/LNGFR pathway in ASD, FXS and autism animal models. However, the activity of sAPPβ may also play a role in brain region overgrowth associated with autism symptoms. Expression of both transthyretin and klotho proteins are upregulated by sAPPβ, but any connection between deficiencies and ASD is still unknown^103^.

To summarize, overall levels of processed APP are increased in FXS with autism symptoms but not idiopathic autism vs. controls. Notably, the relationship between α- and β-secretase pathway processing is significantly altered in idiopathic autism vs. controls, indicating additional APP-related pathways in “anabolic” effects associated with idiopathic autism are not explained simply by mGluR5-based disruption of APP expression. This would not account for the *reduction* in the Aβ levels we observed. On the other hand, FMRP/mGluR5 modifying APP mRNA translation may be sufficient to explain the anabolic effect in FXS. This divergence may point toward other important molecular and clinical differences between FXS and idiopathic autism, particularly in autism without cognitive deficit.

Without the FMRP “brake,” enhanced mGluR5 signaling in FXS individuals would favor excessive total APP, inevitably leading to higher levels of both sAPPα and Aβ levels. Our results regarding sAPP and Aβ peptides in FXS plasma and brain tissue, support some sort of APP-driven anabolic pathway for neurodevelopmental disorders. However, ASD and FXS are also distinct in our sample. Both plasma and brain from ASD and FXS diverge in APP processing, regardless of overall APP protein levels. Significant differences in levels of a neurobiologically important protein or peptide between FXSASD and FXS need not come as a surprise when evaluated in light of both clinical and anatomical comparisons of the two conditions. Such findings provide a rationale to evaluate APP and Aβ-altering/lowering agents (such as posiphen^104^) in treatments for FXS or ASD, and which would be appropriate for one vs. the other.

Biomarker-based investigation is not always fruitful, as shown by the recent failure of mavoglurant clinical trials^104^. Mavoglurant is an mGluR antagonist that had what would be considered ideal results in reversing neurobiological and behavioral symptoms in mouse models. Early clinical trials were also promising. However, a larger and better-powered study resulted in no significant differences between mavoglurant and placebo in any group of the study^105^. Further, a recent mGluR-based therapeutic trial conducted by Roche (RG7090) failed to meet primary and secondary endpoints in treating patients with FXS^106^. Some have begun to conclude that this “calls into question” the mGluR5 model of FXS^105^. We would not go quite so far, as mGluR5 may be an important factor, albeit not the sole determinant, in the development of FXS symptoms. Likewise, arresting mGluR5 activity in adolescent and adult FXS patients may be an example of “closing the barn door after the horse gets out”. Reversal of mechanisms that lead to a disorder usually diagnosed in childhood may have no effect at all if one waits until adulthood. Symptomatic relief at “late” age may work better via “broad-spectrum” psychiatric drugs, such as treatment with lithium showing improvements in several behavioural scales (Autism Behavior Checklist part C: ABC-C; Vineland Adaptive Behaviour Scale: VABS; and Repeatable Battery for the Assessment of Neuropsychological Status: RBANS)^93^. The broad-spectrum antibiotic minocycline may have specific effect vs ASD-associated irritability^93^. Thus, failure of exclusively mGluR-based therapeutic strategies opens the door for considering APP-based therapies, particularly as part of a multi-target approach (e.g., APP levels and/or processing plus mGluR), rather than attempting to find a “magic target” that individually satisfies all criteria.

Thus, we suggest that our findings indicate potentially useful clinical investigation, and unsuccessful clinical trials may inform further work on different molecular pathways. One weakness of the mavoglurant study, mentioned by its authors, was its restriction to adolescents and adults. Given the early developmental function of at least some APP metabolites, unidirectional early-life effects are highly likely in our model. If critical neurological features of ASD and FXS are instilled early in life and depend upon the overall genomic/proteomic milieu of specific developmental stages, altering biochemistry in adolescence or adulthood may be too late.

Such limitations in mind, should overexpression of APP be a fundamental neurochemical dysfunction in FXS (or potentially ASD), several potential drugs may modulate levels of APP protein, such as posiphen and memantine^104^,^107^,^108^,^109^. Should excess specific α-secretase processing of APP contribute significantly to symptoms of ASD independent of overall APP levels, ADAM protein inhibitors may be effective. Some ADAM inhibitors have already shown tolerability and safety in clinical trials. In particular, pioglitazone, currently prescribed to reduce systemic insulin resistance in human subjects, also reduces TACE activity in human muscle^110^. Neurological implications for pioglitazone have already been found in its interaction with apoE gene (*APOE*) genotype vs. phosphorylation of microtubule-associated protein τ (MAPT)^110^,^111^. MAPT hyperphosporylation is present in multiple neurodegenerative disorders, including AD^112^. In addition, bidirectional associations have been found between metabolic syndrome and multiple neurological disorders, including ASD^113^. Other TACE (ADAM10 and ADAM17) inhibitors have also shown safety and tolerability through Stage II clinical trials^113^,^114^. In summary, several recent mega-sequencing studies suggest a molecular link between ASD and FXS, despite their well-attested symptomatic differences. Elucidation of the shared neurobiology of these two neurodevelopmental disabilities is expected to identify novel disease biomarkers and therapeutic targets, which are critical needs for the field. The results presented here have significant mechanistic and translational relevance that support the contribution of APP metabolites to disease phenotypes in ASD and FXS. These outcomes would support utilizing APP metabolite profiles as blood-based biomarkers in clinical trials as well as testing drugs that modulate APP processing as potential therapeutics.

## Methods

The present plasma and brain tissue study (#0211-01) was approved by the Indiana University Purdue University at Indianapolis, USA (IUPUI) Institutional Review Board (IRB). The methods utilized in this study were carried out in accordance with IRB approved guidelines. Further, the use of human subjects was approved by the IUPUI IRB and written informed consent was obtained from parents of all living human subjects. Whenever possible by age and cognitive ability, written assent was also obtained for all live human participants. IUPUI IRB waived informed consent involving brain tissue samples (all *post mortem*). Only de-identified brain tissue samples were utilized. Plasma samples were obtained from 18 typically developing volunteers, 18 FXS subjects, and 20 subjects diagnosed with ASD (Table 1). Briefly, blood samples were drawn in the morning, with plasma-collection tubes containing EDTA. EDTA-incorporated blood samples were centrifuged at 1000 g for 10 minutes at 4 °C. Isolate plasma (top layer) was careful removed and transfered to 1ml microcentrifuge tubes. Tubes were further centrifuged at 4 °C for 12 minutes to ensure complete removal of platelets. Platelet-free plasma was aliquoted and stored at −80 °C for further assays. Children with ASD (n = 20) and FXS (n = 18) met DSM-IV criteria, scored >30 on the Children’s Autism Rating Scale (CARS), and, where available, scored >20 on ADI-R. Children with ASD tested negative for FXS polymerase chain reaction (PCR), urine and plasma for organic and amino acids, chromosomes with microarray, and showed no clinical signs of neurofibromatosis or tuberous sclerosis. Further, all FXS children were FXS PCR positive and, when available, their full mutation was confirmed by *FMR1* DNA test. Age matched, typically developing control subjects (n = 18) had no clinical ASD, CARS scores <30, and were performing normally in school. Equal volumes of the plasma samples were used to determine levels of secreted APPα (sAPPα), secreted APPβ (sAPPβ), and Aβ peptides (both Aβ40 and Aβ42). For all ELISAs linearity of signals were initially carried out with different volumes of diluted raw plasma.

Post mortem left BA10/21 regions (Table 2) were obtained from the Autism Tissue Program (ATP) and the University of Maryland Brain Bank (UMB). The frozen tissues were processed to isolate the secreted fraction as previously described^115^,^116^. Briefly, tissues were homogenized using a mortar and pestle in a “non-detergent” buffer containing 50 mM NaCl and 1% diethylamine (DAE). The homogenates were centrifuged at 30,000g for 4 h (i.e. equivalent of 100,000 g for 1 h) and the supernatant, which is the secreted fraction, was collected and subjected to the ELISA analyses^15^. Data were analyzed statistically from two experiments after normalization to the concentration of the total sample. The FXS portion of our brain sample set had a low number of subjects. This is due to the rarity of the condition and inaccessibility of well-characterized brain tissue specimens, which has been further complicated by the recent breakdown of Harvard Tissue Bank freezer^117^. To partially address this we pseudo replicated the FXS subset by sampling from two different brain regions (BA10 and BA21) vs one region (BA10) for controls and ASD. This was modeled with a generalized linear mixed model (glmm), with “person” and “experiment” as random effects to account for pseudo replication.

Determination of biomarker levels was carried out in plasma using sensitive and specific ELISA (IBL America, Minneapolis). sAPPα levels (pg/ml) were normalized by the total protein content of the plasma. sAPPβ levels were measured in plasma samples from control and FXS subjects using sensitive and specific ELISA (IBL America, Minneapolis). sAPPβ (pg/ml) values were normalized by total protein content of plasma. Aβ40 levels were measured in plasma samples of control and FXS subjects by sensitive and specific ELISA (Wako Pure Chemical Industries, Osaka, Japan). The pg/ml values of Aβ40 were normalized by the total protein content of the plasma samples. Aβ42 levels were measured in plasma samples of control, and FXS subjects by sensitive and specific ELISA (Wako, Osaka, Japan). The pg/ml value of Aβ42 was normalized by the total protein content of the plasma samples. In brain samples, sAPPα, total sAPP, diethylamine (DEA)-extracted Aβ, and soluble fraction Aβ were analyzed by ELISA. Attempt was made to detect Aβ42 by ELISA, without any signal above background without results. We attribute this to sample extraction issues. The Aβ40 and Aβ42 ELISA kits are designed with specific antibodies to each end of the respective peptides, detecting full-length products, only. Truncated Aβ peptides would not have been detected.

Statistical analyses were performed via generalized linear models (glm) or generalized linear mixed models (glmm), followed by simultaneous multiple comparison of diagnostic group means where appropriate. ELISA results were adjusted for total protein content, Western blot for β actin densitometric signal on the same blot. All models were run vs. diagnosis, with and without age as covariate. Models with and without age covariate were compared via second-order Akaike Information Criterion (AICc)^35^, accepting the model with the lower AICc. Models were tested for residuals outliers by Bonferroni-adjusted outlier test. Modeling was repeated excluding the most extreme outlier that passed this test. Standardized effect sizes (Hedge’s *g* or ω^2^)^118^,^119^ were also calculated.

## Ethics statement. 

The present plasma and brain tissue study (#0211-01) was approved by the Indiana University Purdue University at Indianapolis, USA (IUPUI) Institutional Review Board (IRB). The methods utilized in this study were carried out in accordance with IRB approved guidelines. Further, the use of human subjects was approved by the IUPUI IRB and written informed consent was obtained from parents of all living human subjects. Whenever possible by age and cognitive ability, written assent was also obtained for all live human participants. Although IUPUI IRB waived informed consent involving brain tissue samples (all *post mortem*), we still obtained written parental consent for use of tissues from minor children. Only de-identified brain tissue samples were utilized.

## Additional Information

**How to cite this article**: Ray, B. *et al*. Finding novel distinctions between the sAPPα-mediated anabolic biochemical pathways in Autism Spectrum Disorder and Fragile X Syndrome plasma and brain tissue. *Sci. Rep.* **6**, 26052; doi: 10.1038/srep26052 (2016).

## Supplementary Material

Supplementary Information

## Figures and Tables

**Figure 1:**
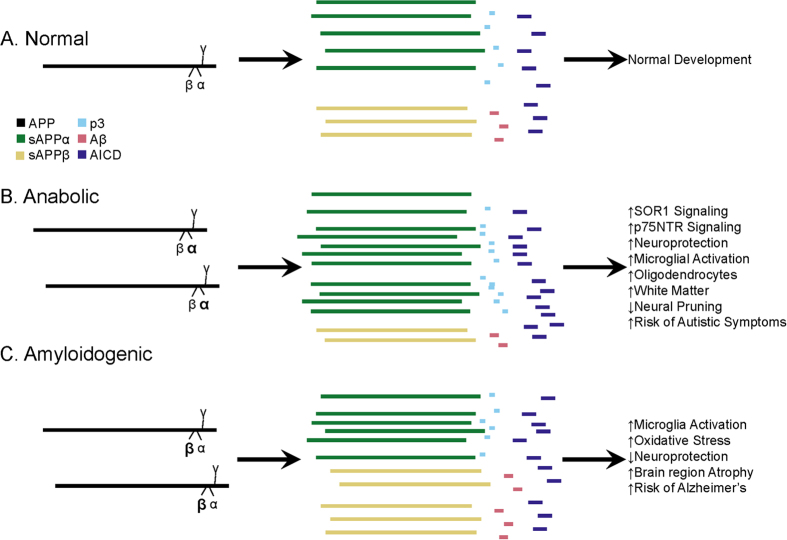
Contrast of normal and pathogenic APP protein processing pathways. (**A**) Normal pathway. APP protein (green) is processed by secretases (violet) and usually cleaved at the α-secretase site by ADAM10 or ADAM17 (α), then afterwards by the γ-secretase complex (γ). This produces sAPPα, the non-pathogenic p3 peptide product, and the APP intracellular domain (AICD). The sAPPα product is both neuroprotective and activates microglia. A minority of APP is instead cleaved by BACE1 (β) before γ cleavage to produce sAPPβ, Aβ, and AICD. These balanced processes, under normal conditions, lead to neural pruning and normal development at appropriate times. This is the majority APP processing pathway. (**B**) Anabolic dysfunction (pro-autistic). Overproduction of APP may be accompanied by excess α-secretase activity, resulting in overproduction of sAPPα, which both activates neuroglia and is neuroprotective. Neuroprotective activity would presumably overwhelm microglial activation, since other molecules, presumably not also over-produced in this scenario, also activate microglia during neural pruning and would not be able to make up the difference to overwhelm additional sAPPα neuroprotection. The net result would bring about neuronal overgrowth and risk for ASD. Some element would also play a role in FXS symptoms. (**C**) Amyloidogenic (pro-Alzheimer’s). This pathway is associated with neurodegeneration and Alzheimer’s disease. Possible increase in APP protein and BACE1 protein levels (β) result in increased cleavage at the β-secretase site and then by γ-secretase complex (γ). This produces sAPPβ, pathogenic/neurotoxic Aβ peptide, and AICD. sAPPβ activates microglia without offering neuroprotection. Thus, two of the three major products of the β-secretase pathway are neurodegenerative. If the β-secretase pathway becomes excessive, risk for Alzheimer’s disease increases.

**Table 1:** Subject Demographics.

Typical Development			ASD			FXS		
CARS	Age	Ethnicity	CARS	Age	Ethnicity	CARS	Age	Ethnicity
15	5 yr, 10 mo	Caucasian	46	8 yr, 2 mo	Caucasian	37	18 yr, 8 mo	Caucasian
15	4 yr, 6 mo	Caucasian	30	4 yr, 2 mo	Caucasian	23	7 yr, 7 mo	Caucasian
15	3 yr, 9 mo	Caucasian	33	6 yr, 11 mo	Caucasian	55	3 yr, 3 mo	Caucasian
15	3 yr, 7 mo	Caucasian	36	4 yr, 7 mo	Caucasian	43	2 yr, 10 mo	Caucasian
15	5 yr, 2 mo	Caucasian	52	4 yr, 0 mo	Caucasian	22	6 yr, 11 mo	Caucasian
15	3 yr, 1 mo	Caucasian	42	5 yr, 3 mo	Caucasian	42	3 yr, 2 mo	Caucasian
15	5 yr, 11 mo	Hispanic	30	5 yr, 2 mo	Caucasian	45	17 yr, 9 mo	Caucasian
15	11 yr, 10 mo	Caucasian	51	5 yr, 1 mo	Caucasian	40	21 yr, 2 mo	Caucasian
15	14 yr, 9 mo	Caucasian	33	6 yr, 2 mo	Caucasian	29	8 yr, 8 mo	Caucasian
15	18 yr, 3 mo	Caucasian	48	4 yr, 4 mo	Caucasian	43	3 yr, 11 mo	Hispanic
18	11 yr, 3 mo	Caucasian	32.5	3 yr, 7 mo	Caucasian	56	8 yr, 7 mo	Caucasian
15	9 yr, 7 mo	Caucasian	43	3 yr, 10 mo	Caucasian	33	6 yr, 0 mo	Caucasian
15	13 yr, 7 mo	Caucasian	47	4 yr, 3 mo	Caucasian	42	7 yr, 0 mo	Caucasian
15	3 yr, 10 mo	Caucasian	37	3 yr, 7 mo	Caucasian	32	6 yr, 8 mo	Caucasian
15	7 yr, 7 mo	Caucasian	43	5 yr, 9 mo	Caucasian	37	6 yr, 11 mo	Caucasian
15	2 yr, 8 mo	Caucasian	39	7 yr, 6 mo	Caucasian	37	11 yr, 6 mo	Caucasian
15	2 yr, 10 mo	Caucasian	39	2 yr, 8 mo	Caucasian	51	13 yr, 1 mo	Caucasian
15	8 yr, 11 mo	Caucasian	39	5 yr, 11 mo	Caucasian	32	6 yr, 1 mo	Caucasian
			56	6 yr, 1 mo	Caucasian			
			45	3 yr, 1 mo	Caucasian			

**Table 2:** Brain Tissue Demographics.

ID	Diagnosis	Age	PMI	Gender	Brain Location	
*AN03217*	*Control*	*19*	*18.58*	*M*	*BA 20*	
*AN17425*	*Control*	*16*	*26.16*	*M*	*BA 21*	
*AN02456*	*Control*	*4*	*NA*^a^	*F*	*BA 21*	
*AN07444*	*Control*	*17*	*30.75*	*M*	*BA 21*	
*UMB4722*	*Control*	*14*	*16*	*M*	*BA 21*	
*UMB1860*	*Control*	*8*	*5*	*M*	*BA 21*	
*UMB5319*	*Control*	*8*	*17*	*M*	*BA 21*	
*UMB5180*	*Control*	*1*	*25*	*M*	*BA 21*	
*UMBM3228M*	*Control*	*11*	*20*	*M*	*BA 21*	
*1148*	*Control*	*84*	*NA*	*F*	*BA 21*	
*1221*	*Control*	*81*	*NA*	*M*	*BA 21*	
*AN01570*	*ASD*	*18*	*6.75*	*F*	*BA 21*	
*AN02987*	*ASD*	*15*	*30.83*	*M*	*BA 21*	
*AN03345*	*ASD*	*2*	*4*	*M*	*BA 21*	
*AN08873*	*ASD*	*5*	*25.5*	*M*	*BA 21*	
*AN16115*	*ASD*	*11*	*12.88*	*F*	*BA 21*	
*AN16641*	*ASD*	*9*	*27*	*M*	*BA 21*	
*AN19511*	*ASD*	*8*	*22.16*	*M*	*BA 21*	
*UMB4806b*	*FXS*	*9*	*22*	*M*	*BA 21, BA 10*	
*UMB5319c*	*FXS*	*71*	*NA*	*M*	*BA 21, BA 10*	
*UMB4751d*	*FXS*	*21*	*5*	*M*	*BA 21, BA 10*	

^a^NA: Not available. ^b^FMR1 gene deleted. ^c^Xq27.3 abnormal karyotype. ^d^Premutation CGG repeats = 88.
