# Supplementary material for: Finding novel distinctions between the sAPPα-mediated anabolic biochemical pathways in Autism Spectrum Disorder and Fragile X Syndrome plasma and brain tissue
Source: Sci Rep. 2016 May 23;6:26052. doi: 10.1038/srep26052 (PMC4876513; doi:10.1038/srep26052)
Supplement: Supplementary Information [file srep26052-s1.pdf]

Finding novel distinctions between the sAPPα-mediated anabolic biochemical pathways in Fragile X Syndrome and idiopathic Autism plasma and brain tissue

Balmiki Ray, Deborah K. Sokol, Bryan Maloney, & Debomoy K. Lahiri

Supplemental Material: Age covariate

Age was available for all subjects. Brain models were run with and without age as a covariate. In all cases except ADAM17, these produced weaker models, as measured by second-order Akaike information criterion. These models are presented herein. FXS samples had to be excluded from the analysis due to small N vs. the number of factors and interactions. Levels of sAPPα, total sAPP, and the ratio of sAPPα to total sAPP had no significant differences for either factor or for the diagnosis × age interaction (A-C). Aβ may be more interesting. DEA-extracted (membrane-bound) Aβ40 showed a distinct interaction between diagnosis and age. Specifically, while DEA-extracted Aβ40 did not change with age among control subjects, it decreased with age in ASD, although neither age nor diagnosis were significant by itself in this model. Conversely, while soluble Aβ40 decreased with age in control subjects, it remained steady in ASD. However, while difference by diagnosis was significant, the interaction with age was not. Total Aβ40 did not show significant differences in the two-way model by diagnosis, age, or their interaction. These models may be interesting particularly if contrasted against the ADAM17 model. Unfortunately, levels of BACE1 (β-secretase), and the other major α-secretase proteins (ADAM9, ADAM10) were not measured, preventing a more complete overall picture. Likewise, levels of Aβ42 were not available for analysis. In addition, these models were rejected on the basis of higher AICc (). AICc expresses the Kullback-Liebler divergence, which approximates the information lost in a given model vs. its “fit”, taking into account number of terms and sample size. AICc for the two-way models was >2 higher than for diagnosis-only models. With a larger sample size, stronger models may be possible with covariates.

Finding novel distinctions between the sAPPα-mediated anabolic biochemical pathways in Fragile X Syndrome and idiopathic Autism plasma and brain tissue

Balmiki Ray, Deborah K. Sokol, Bryan Maloney, & Debomoy K. Lahiri

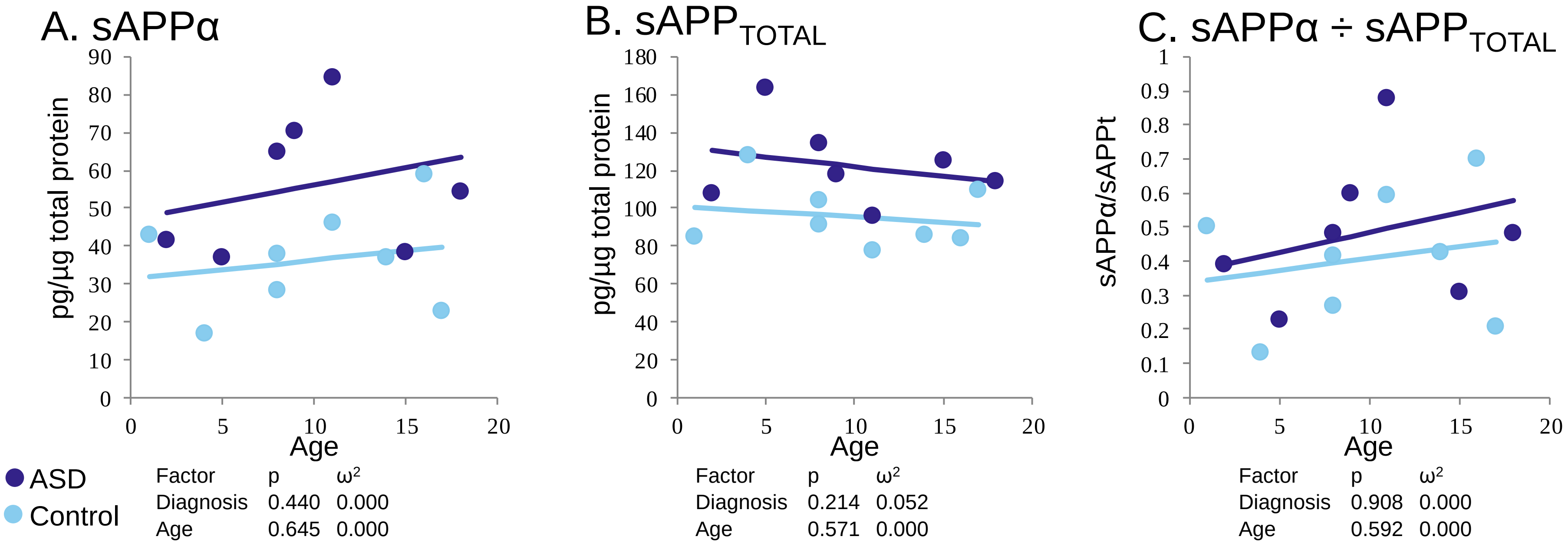

Factor	p	ω^2
Diagnosis	0.440	0.000
Age	0.645	0.000
Diag × Age	0.851	0.000

Factor	p	ω^2
Diagnosis	0.214	0.052
Age	0.571	0.000
Diag × Age	0.887	0.000

Factor	p	ω^2
Diagnosis	0.908	0.000
Age	0.592	0.000
Diag × Age	0.842	0.000

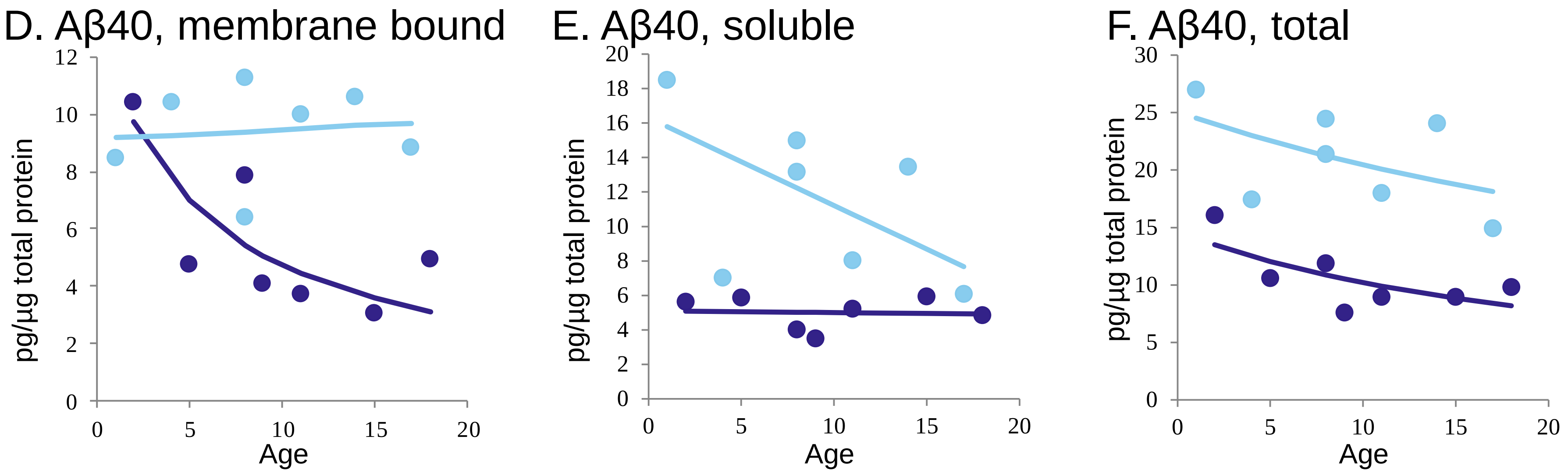

Factor	p	ω^2
Diagnosis	0.340	0.000
Age	0.019	0.248
Diag × Age	0.021	0.239

Factor	p	ω^2
Diagnosis	0.003	0.504
Age	0.899	0.000
Diag × Age	0.155	0.047

Factor	p	ω^2
Diagnosis	0.078	0.135
Age	0.058	0.169
Diag × Age	0.211	0.037
